# What to feed or what not to feed-that is still the question

**DOI:** 10.1007/s11306-021-01855-7

**Published:** 2021-11-20

**Authors:** James C. Lech, Sophia I. Dorfsman, Zoltán Répás, Tjaart P. J. Krüger, Ingrid Melinda Gyalai, László G. Boros

**Affiliations:** 1 Faculty of Science, Vrije Universiteit Amsterdam, Amsterdam, The Netherlands; 2 National Research Foundation, Pretoria, South Africa; 3 International EMF Project & Optical Radiation, World Health Organization, Pretoria, South Africa; 4 Department of Radiology and Nuclear Medicine, Academic Medical Center, University of Amsterdam (UMC), Amsterdam, The Netherlands; 5 University of Gastronomic Sciences, Bra, Italy; 6 Institute of Food Science, Faculty of Agricultural and Food Sciences and Environmental Management, University of Debrecen, Debrecen, Hungary; 7 Department of Physics, University of Pretoria, Pretoria, South Africa; 8 Forestry and Agricultural Biotechnology Institute (FABI), University of Pretoria, Pretoria, South Africa; 9 Faculty of Agriculture, University of Szeged, Hódmezővásárhely, Hungary; 10 SiDMAP, LLC and the Deutenomics Science Institute, Los Angeles, CA USA

**Keywords:** Grass feeding, Total mixed ration, Mitochondria, Branched chain amino acids, Deupletion, Deutenomics

## Abstract

**Introduction:**

This review addresses metabolic diversities after grain feeding of cattle using artificial total mixed ration (TMR), in place of pasture-based feeding.

**Objectives:**

To determine how grain feeding impairs the deuterium-depleting functions of the anaplerotic mitochondrial matrix during milk and meat production.

**Methods:**

Based on published data we herein evaluate how grain-fed animals essentially follow a branched-chain amino acid and odd-chain fatty acid-based reductive carboxylation-dependent feedstock, which is also one of the mitochondrial deuterium-accumulating dysfunctions in human cancer.

**Results:**

It is now evident that food-based intracellular deuterium exchange reactions, especially that of glycogenic substrate oxidation, are significant sources of deuterium-enriched (^2^H; D) metabolic water with a significant impact on animal and human health. The burning of high deuterium nutritional dairy products into metabolic water upon oxidation in the human body may contribute to similar metabolic conditions and diseases as described in state-of-the-art articles for cows. Grain feeding also limits oxygen delivery to mitochondria for efficient deuterium-depleted metabolic water production by glyphosate herbicide exposure used in genetically modified crops of TMR constituents.

**Conclusion:**

Developments in medical metabolomics, biochemistry and deutenomics, which is the science of biological deuterium fractionation and discrimination warrant urgent critical reviews in order to control the epidemiological scale of population diseases such as diabetes, obesity and cancer by a thorough understanding of how the compromised metabolic health of grain-fed dairy cows impacts human consumers.

## Introduction

Metabolomics approaches provide excellent resources to assist producers and consumers via improving nutritional efficiency in animals and humans, thereby enhancing their well-being without negatively impacting the environment. Some of the excellent contributions in the field (see, e.g., Adewuyi et al., 2005; García-Roche et al., 2019) specifically address metabolic diversities in glucose and fatty acid metabolism found in lactating cows in systems that involve either a total mixed ration (TMR) or pasture-based feeding strategies. According to western industry standards the TMR consists of crushed corn plant (forage), alfalfa, barley, corn, sunflower, and soybeans (University of South Dakota). Corn, alfalfa and soy could be grown from herbicide glyphosate-resistant plant seeds and treated with glyphosate during growth, while barley and sunflowers are often sprayed with glyphosate before harvest as a desiccant. The authors used various analytical methods to decipher intermediary flux via metabolite measurements that reflected on biochemical reaction architectures. The diverse mitochondrial adaptation to TMR and pasture-based feeding was recently strengthened by genetic and protein expression experiments (García-Roche et al., 2021). These are clearly substantial advancements for the field to understand the significance of biochemical reactions required for the production of milk and other biosynthetic products in cattle fed either by grain or grass. Detailed understanding of adaptive metabolite flux architectures to cow-feeding protocols is important because the glycogenic substrates used by grain-fed animals such as corn starch, branched-chain amino acids, and odd-chain fatty acids have inherently higher heavy hydrogen isotopic (deuterium) contents (Schleucher et al., 1999), which have been associated with cellulo-proliferative disorders (Boros et al., 2016, 2017). The rapid and robust metabolic exchange of deuterium in various cellular water compartments from deuterated starch-deriving glucose via its glycogenic products and precursors has been described in the medical literature (Ben-Yoseph et al., 1994) with recent updates using magnetic resonance imaging (MRI) methods (Mahar et al., 2021). Those mechanisms are historically related to a dose-dependent damage to mitochondrial ATP synthase in the heart muscle of cows in response to deuterium (Dorgan & Schuster, 1981; Urbauer et al., 1984). Such damage precisely occurs by the biological effects of deuterium on mitochondrial ATP synthase by deuteronation (Olgun, 2007), which is the process of replacing a proton with a deuterium atom anywhere in life. Deuteronation is common in metabolically challenged cow products, dairy and meat alike, after TMR feeding that may be of concern also to human health at the consumer end, as described below. Our review points to important developments in deutenomics because, according to the United States F.D.A. website, more than 95% of animals used for meat and dairy in the United States eat GMO crops including alfalfa, canola, corn, and soy (Regulated Products, 2020). Deutenomics is the interdisciplinary approach to medical biochemistry as deuterium introduces large sub molecular isotope effects as a rapidly evolving science with trajectories into diagnostics, prevention, as well as clinical interventions.

### Biochemical impact of various feeding protocols

According to the two animal nutrition articles by Garcia-Roche to open such insights with an edge to formulate opinions, Holstein–Friesian cows were fed either by artificially mixed grain-/protein-based fodder or grass from natural pasture for longer periods (over 3 months) after calving. These articles outstandingly explore many protein, glucose, and fatty acid metabolism pathways by mapping circulating and tissue-bound intermediates, enzyme reactions, and hormonal changes. Milk, blood and liver samples were taken regularly for detailed analysis of metabolic products, hormones, gene expression (transcription), and protein studies (translation).

**Fig. 1:**
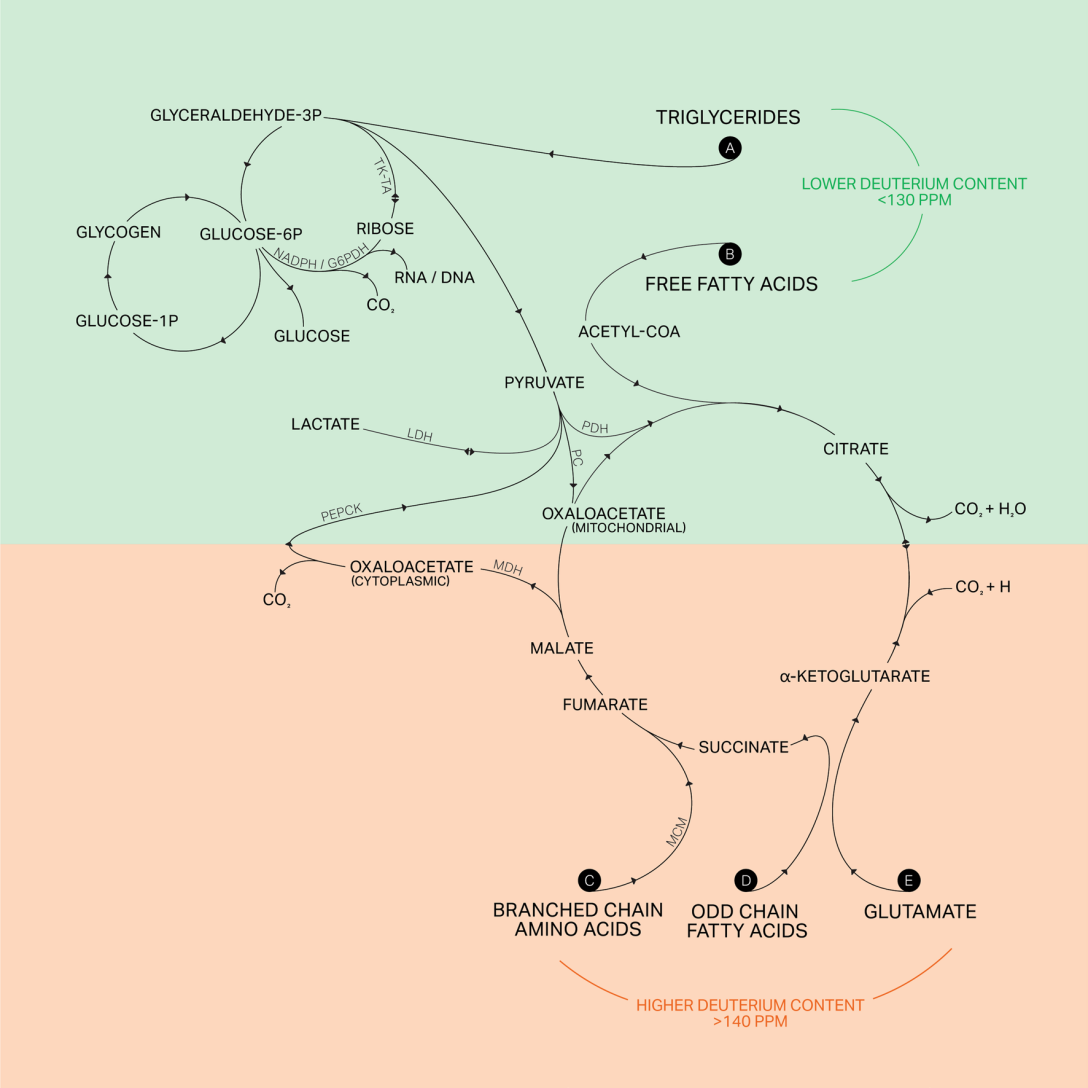
Significantly different biochemical reaction architectures for the processing of deuterium-rich and -poor feedstocks under artificial TMR cattle feed (orange) and grass (green) feeding strategies in dairy cows, respectively. The text frames labelled **A** and **B** show the ketogenic substrate metabolites of grass-fed animals that lead to the combustion of naturally lower deuterium fats, glycerol and fatty acids that produce deuterium-depleted (deupleted) matrix water for mitochondria. Substrates labelled **C** and **D** show essential branched-chain amino acid feedstocks of artificially TMR fed animals, such as leucine, isoleucine and valine, as well as methionine, threonine and thymine, and their odd chain fatty acid derivatives with higher deuterium content, as explained in the article. Reaction path labelled **E** shows the use of higher deuterium glutamine by reductive carboxylation towards branching mitochondrial function. Please note that glutamate and its derivative ketoglutarate (alpha-carbon) is also used via oxidation to produce NADPH, i.e. the reducing equivalent for reductive carboxylation towards citrate production in severely defective mitochondria of grain-fed cows (Mullen et al., 2014). Artificial TMR cattle feeds used at Western farming establishments also contain glyphosate. *PC *Pyruvate Carboxylase, *MCM *Methylmalonyl-CoA Mutase (also known as isomerase), *G6PDH *Glucose-6-Phosphate Dehydrogenase, *TK *Transketolase, *TA *Transaldolase, *NADPH *Nicotine Adenine Dinucleotide Phosphate (protonated), *LDH *Lactate Dehydrogenase, *PDH *Pyruvate Dehydrogenase, *MDH *Malate Dehydrogenase, *PEPCK *Phosphoenolpyruvate Carboxykinase

**Grass-fed animals** showed ketogenic metabolism based on circulating fats and fatty acids with an inherently lower deuterium content (< 130 ppm) in various plant fatty acid products (Billault et al., 2005) (substrates labeled A and B in Fig. 1). It is also important that messenger-RNA expression of gluconeogenic enzymes for the formation of circulating glucose was significantly increased. These enzymes are involved in the conversion of a portion of grass-based low deuterium precursor glycerol to glucose (Schleuche et al., 1999). Besides its conjugated fatty acids, plant-derived glycerol is naturally low in deuterium, and glycerol is a significant gluconeogenic precursor after its phosphorylation in the liver by glycerol kinase of cows. Grass-derived glycerol, which is the source of pyruvic acid, can also feed full catabolic and deuterium-depleting (deupleting) TCA cycle (tricarboxylic acid-, Krebs-Szent-Györgyi cycle) functions via citrate synthesis and deuterium depleted (deupleted) matrix water production and recycling. Such deupleting metabolic adaptation protects mitochondrial ATPase and other ion pump related nanomotor functions during substrate transport, oxidation and anaplerosis necessary for net (new) biomolecule synthesis during lactation (Fig. 1).

On the other hand, in **artificially mixed grain-fed animals**, appreciative amounts of branched-chain amino acids (leucine, isoleucine and valine) with higher (> 140 ppm) deuterium content (Lecchi & Abramson, 2000) enter the TCA cycle via succinic acid that triggers methylmalonyl-CoA mutase gene expression in mitochondria. It is apparent from the papers of Garcia-Roche that grain feeding switches cows to the burning of high-deuterium carbohydrates (Schleucher et al., 1999), branched-chain amino acids as well as odd-chain fatty acids formed from them based on the composition of TMRs (University of South Dakota) (substrates labeled C and D in Fig. 1). Another disadvantage is the higher need for glutamine used by reductive carboxylation (Holleran et al., 1995) with irreversible branching towards citrate and fatty acid synthesis from an amino acid (glutamine) source, which reverses the direction of the TCA cycle from a deuterium-depleting function to a deuterium-accumulating mode of operation (Fig. 1, input metabolic substrate marked as E).

In natural **grass-fed animals**, the Krebs-Szent-Györgyi cycle is supplied by deuterium-depleted glycerol- and pyruvate-deriving anaplerotic oxaloacetic acid by the enzyme pyruvic acid carboxylase, which produces conventionally the first product of the cycle, citric acid by its synthase, which uses mitochondrial deuterium-depleted water (DDW) as a source of metabolic protons during catabolic reactions downstream.

Thus, from the excellent publications of Garcia-Roche described above we easily learn how grain feeding diverts biochemical processes from using low-deuterium natural ketogenic, i.e., fatty acid beta carbon product based substrates that can be traced alongside natural grazing. Low deuterium grass-based substrate oxidation seeks to provide lower deuterium levels in the body products of grass-fed cattle under natural metabolic ketosis. In contrast, artificially mixed GMO grain and soy are richer in carbohydrates and proteins and induce enzymes that use branching amino acids and odd-chain fatty acids, both of which are prone to result in heart failure (Sun et al., 2016), diabetes, and obesity (Halama et al., 2016). When grain-fed cows produce organic molecules to make milk and meat in an environment with elevated deuterium over several months of lactation, like those studied in this article, their metabolic adaptation reveals an undesired flux distribution as a system property to cellular metabolism (Fig. 1). These recently uncovered crosstalks among branched-chain amino acid and odd-chain lipid metabolism during adipogenesis in grain-fed cows might contribute to a better understanding of the molecular mechanisms of obesity and diabetes, which may have potential implications in clinical predictions and population disease trends. We believe that the potentially high deuterium content of many processed dairy products (school milk, cheeses, sour cream, kefir, meat, and interior fats), originating from grain-fed cattle, needs to be described along this excellent article (García-Roche et al., 2021), as a significant contributing factor to a number of metabolic and degenerative disease conditions, such as cancer, obesity, diabetes, and Alzheimer’s, which affect multiple age groups at the same time.

Another **significant difference** between the food sources for pasture-raised cows versus grain-fed cows is the **amount of glyphosate** contamination in the feed. Glyphosate is an artificial glycine -derivative herbicide that is widely used in grain production, and it has known toxic effects on critical enzymes involved in the maintenance of nicotinamide adenine dinucleotide phosphate (NADP^+^), which, in turn, is critical for deuterium homeostasis. Dairy cows in Denmark, for example, are highly exposed to glyphosate in their feed, and it impacts their metabolism (Krüger et al., 2013). The severely defective mitochondria of grain-fed cows are also likely due to toxic glyphosate exposures that collide with the higher deuterium contents in their feed, alike, as research has shown that glyphosate severely damages mitochondria (

Bailey et al., 2018). The above is further supported by glyphosate to block the shikimate pathway in both plants and microbes, from where tryptophan, a product of the shikimate pathway, is an essential precursor to NAD (Lu et al., 2013). Glyphosate's influence on both plant-based food sources and gut microbes likely causes deficiencies in NAD with the far-reaching mitochondrial issues described by García-Roche et al. (2019, 2021) in grain-fed lactating cows. Glyphosate, for example, suppresses G6PD, an enzyme that is highly active in red blood cells, which plays an essential role in supplying NADPH to antioxidants such as glutathione in the mitochondria (Cattani et al., 2014). Nicotinamide adenine dinucleotide phosphate is also the essential reducing equivalent during de novo (net) fatty acid synthesis from mitochondrial citrate. Mitochondrial isocitrate dehydrogenase and glutamate oxidation may become active in grain-fed cows in order to resupply NADPH, due to suppressed G6PD activity. Glyphosate has also been shown to suppress succinate dehydrogenase, a critical enzyme in both oxidative phosphorylation and the citric acid cycle (Ugarte, 2014). Additionally, glyphosate not only interferes with the synthesis of the pyrrole ring in heme (Kitchen et al., 1981), but it also inhibits heme’s product, i.e. the hemoglobin of red blood cells to transport oxygen to tissues, thus limiting mitochondrial deuterium-depleted metabolic water production via oxygen deprivation. This occurs because glyphosate shows significant structural homology with glycerol bisphosphate, the substrate of bisphosphoglycerate mutase (BPGM).This enzyme, unique to erythrocytes and placental cells, is responsible for the catalytic synthesis of 2,3-bisphosphoglycerate (2,3-BPG) from 1,3-bisphosphoglycerate that regulates oxygen delivery to tissues. It seems likely that glyphosate's disruption of deuterium homeostasis is one of the most important human disease causing factors distinguishing the metabolic impact of deuterated grain-fed cow products from that of deupleted grass-fed cow products for human consumption with consequent metabolic water production.

## Discussion

From the above, it is clear that grain feeding of cattle, in place of pasture-based feeding, induces mitochondrial deuterium-depletion deficiency and thereby impairs the deuterium-depleting function of the mitochondrial matrix during milk and meat production. Grain-fed animals exposed to elevated deuterium and glyphosate essentially follow a branched-chain amino acid and odd-chain fatty acid-based reductive carboxylation dependent feedstock, which is also one of the significant mitochondrial metabolic features of human cancer cell metabolism (Mullen et al., 2012; 2014). In the context of deutenomics, artificial total mixed ration feeding seriously interferes with the deuterium-depletion process in dairy cows, and the consequent burning of their high deuterium nutritional products into metabolic water upon oxidation in the human body may contribute to similar metabolic conditions and diseases as described for cows above. We acknowledge that there are many additional factors that differ between diets besides their deuterium and glyphosate contents. However, differences in deuterium content and factors that interfere with deuterium depletion between metabolically glycogenic (grain-fed) and ketogenic (grass-fed) animals are gaining interest for translational medicine (Boros et al., 2017) due to deuterium’s exceptional isotopic-substitution effect via the breakdown of collective proton tunneling (Drechsel-Grau & Marx, 2014). This process, i.e. the tunneling of protons amongst recycled structured (bound) metabolic water compartments is the prime source of biological energy produced in the mitochondrial matrix (Ruffle et al., 2002), where tight proton tunneling makes deuterium about a thousand fold more significant inorganic element than it is expected from its relatively low natural abundance. The above invokes full tunneling of all isotopes of hydrogen during hydride ion transfer reactions, with barriers reflecting the heavy atom (^2^H; D) (Klinman, 2006) that necessitates an integration of nutritional data implicating hydrogen tunneling in mitochondrial enzymes in place of the Swain–Schaad relationships and the semi-classical temperature dependence of the hydrogen isotope effect in biology. It is also apparent from data reviewed herein that deuterium content of nutrients has far reaching consequences by potentially explaining hydrogen and deuterium related nuclear quantum effects (Shrestha et al., 2019), such as proton tunneling and delocalization, for sub-molecular medical and agricultural sciences, in close relation with nutrition. With our greater understanding of proton tunneling in sub-molecular biological sciences our review readily points to deuterium related large kinetic isotope effects in physics, inorganic chemistry and translational medicine as a desired interdisciplinary investigative approach to improve public health.
